# Case report: Anlotinib plus oral etoposide: a potential salvage therapy based on insights from EGFR-TKI-resistant SCLC transformation PDX and clinical settings

**DOI:** 10.3389/fonc.2025.1661273

**Published:** 2025-10-08

**Authors:** Zixi Wang, Xiaoyu Li, Lili Jiang, Weiya Wang, Kai Xiao, Ting Zhang, Li Li, Dan Li, Yongsheng Wang

**Affiliations:** ^1^ Division of Thoracic Tumor Multimodality Treatment, Cancer Center, West China Hospital, Sichuan University, Chengdu, Sichuan, China; ^2^ Clinical Trial Center, National Medical Products Administration Key Laboratory for Clinical Research and Evaluation of Innovative Drugs, West China Hospital, Sichuan University, Chengdu, Sichuan, China; ^3^ Department of Pathology, West China Hospital, Sichuan University, Chengdu, Sichuan, China; ^4^ Precision Medicine Research Center, Sichuan Provincial Key Laboratory of Precision Medicine, National Clinical Research Center for Geriatrics, Frontiers Science Center for Disease-Related Molecular Network, West China Hospital, Sichuan University, Chengdu, Sichuan, China; ^5^ Laboratory of Nonhuman Primate Disease Modeling Research, State Key Laboratory of Biotherapy, West China Hospital, Sichuan University, Chengdu, Sichuan, China; ^6^ Institute of Clinical Pathology, West China Hospital, Sichuan University, Chengdu, Sichuan, China; ^7^ Institute of Respiratory Health, Frontiers Science Center for Disease-related Molecular Network, and Precision Medicine Research Center, Precision Medicine Key Laboratory of Sichuan Province, West China Hospital, Sichuan University, Chengdu, Sichuan, China

**Keywords:** non-small cell lung cancer (NSCLC), small cell lung cancer transformation, epidermal growth factor receptor tyrosine kinase inhibitor (EGFR-TKI), patient-derived tumor xenograft (PDX), anlotinib

## Abstract

Small cell lung cancer (SCLC) transformation is one of the resistance mechanisms to epidermal growth factor receptor tyrosine kinase inhibitor (EGFR-TKI) treatment in patients with lung adenocarcinoma (LUAD). Platinum-etoposide chemotherapy is the most common regimen for patients with SCLC transformation. Despite a high initial response rate, patients experience rapid disease progression, and there is a paucity of evidence for further-line therapy. In this study, we report two cases of patients with EGFR 19del-mutated and EGFR-TKI-resistant transformed SCLC who failed with platinum-etoposide chemotherapy. We explored a novel combination strategy of anlotinib and orally administered etoposide in these two patients, guided by the results of a patient-derived tumor xenograft (PDX) model. The combination regimen was clinically applied when platinum-etoposide chemotherapy failed, and both patients benefited from the treatment. To our knowledge, this is the first report of anlotinib plus oral etoposide as a potential salvage therapy for patients with EGFR-TKI-induced SCLC transformation resistant to platinum-based chemotherapy. Both the PDX model and clinical cases support the efficacy of this regimen, providing a promising therapeutic option for patients with SCLC transformation after platinum-etoposide chemotherapy resistance.

## 1 Introduction

Lung cancer is the leading cause of cancer-related mortality worldwide (1). Histologically, non-small cell lung cancer (NSCLC) accounts for approximately 85% cases, whereas small cell lung cancer (SCLC) comprises the remaining 15% (2–4). Notably, SCLC transformation occurs in 3-14% of NSCLC patients who develop acquired resistance to first- and second-generation epidermal growth factor receptor tyrosine kinase inhibitors (EGFR-TKIs) (5). The molecular and phenotypic characteristics of transformed SCLC are similar to those of *de novo* SCLC, but transformed SCLC is associated with worse clinical manifestations, faster progression, and poorer prognosis (6, 7).

The current standard of care (SoC) for patients with extensive-stage SCLC (ES-SCLC) consists of platinum-etoposide chemotherapy combined with an immune checkpoint inhibitor (ICI, such as atezolizumab or durvalumab) (8). In addition, several novel therapeutic strategies are under investigation, including ICI, small molecule targeted therapies, bispecific antibodies, and antibody–drug conjugates, such as tarlatamab and lurbinectedin (3, 4, 8). Similarly, platinum-etoposide chemotherapy is the most commonly used first-line therapy for transformed SCLC; however, treatment responses tend to be transient (9). Anlotinib, a multitarget tyrosine kinase inhibitor, has been approved by the National Medical Products Administration (NMPA) for the third-line treatment of patients with advanced SCLC in 2019. It targets tumor angiogenesis and proliferative signaling, including vascular endothelial growth factor receptor (VEGFR) 1 to 3, platelet-derived growth factor receptor (PDGFR), fibroblast growth factor receptor (FGFR), and c-kit (10, 11). Although platinum-etoposide chemotherapy and anlotinib monotherapy have shown efficacy in patients with transformed SCLC (7), evidence supporting second-line and subsequent treatment strategies remains scarce.

In this paper, we report two cases of patients with transformed SCLC who failed platinum-etoposide chemotherapy but benefited from subsequent treatment with anlotinib and orally administered etoposide, and this regimen was administered based on the results of a patient-derived tumor xenograft (PDX) experiment.

## 2 Case description

### 2.1 Case 1

**Figure 1:**
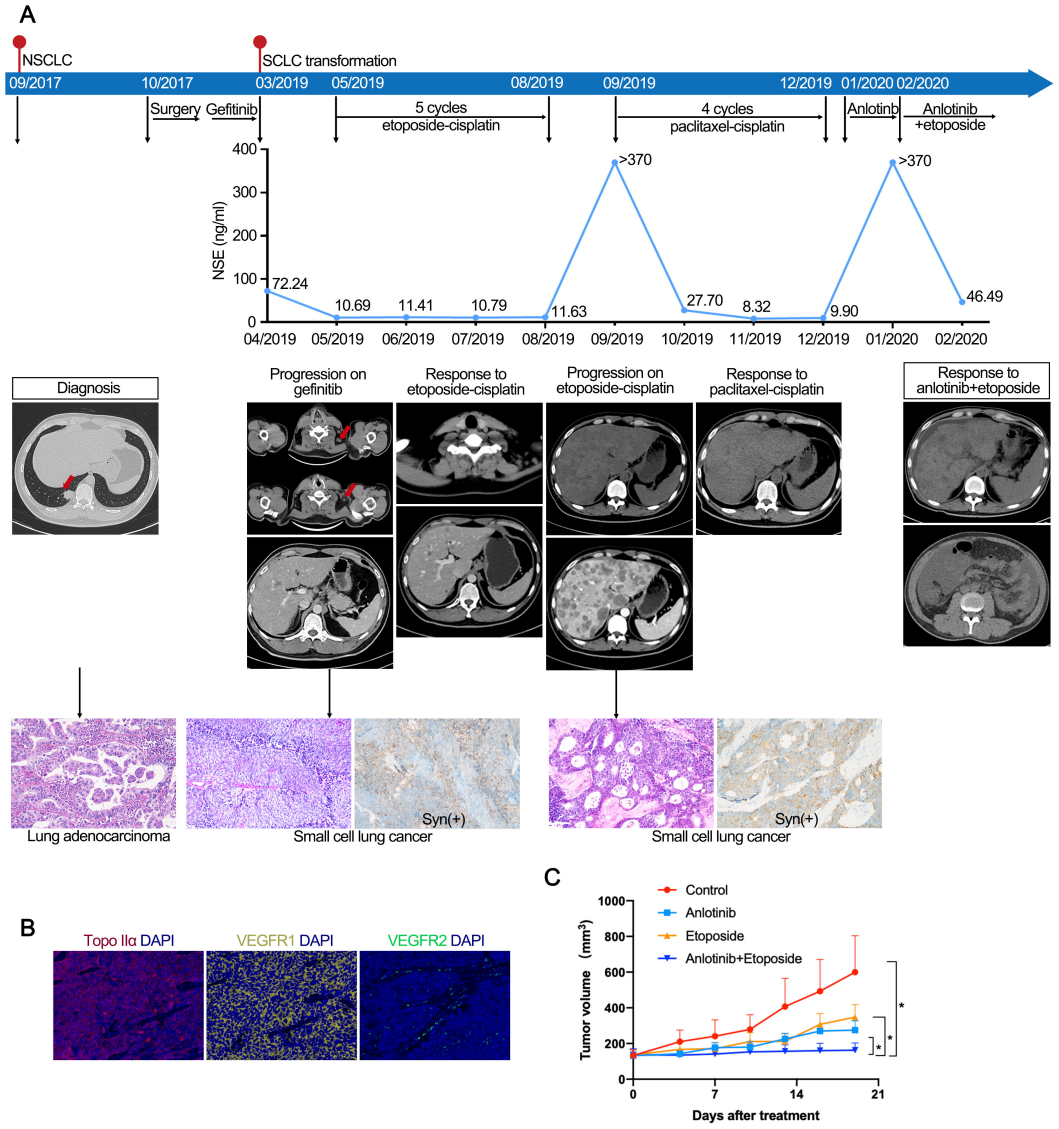
**(A)** Case 1. Case presentation of the transformation of lung adenocarcinoma to small cell lung cancer, including treatment details, available radiographic and pathological findings. **(B)** Immunofluorescence staining of case 1 confirmed the expression of Topo IIα, VEGFR1, and VEGFR2. **(C)** Response to treatment in PDX models. Tumor volume changes of control, etoposide, anlotinib, and anlotinib with etoposide in mice engrafted with PDX tumors (n=5). The PDX model showed superior tumor control with etoposide and anlotinib combined versus single-agent therapy. PDX, patient-derived xenografts.

Recurrent lung cancer developed in a young male nonsmoker with a previous diagnosis of EGFR 19del-mutated lung adenocarcinoma (LUAD) (pT2N1M0, stage IIB), after curative surgery and 15 months of gefitinib treatment (**Figures 1A, B**). At the time of disease progression, the biopsy revealed SCLC transformation, while next-generation sequencing (NGS) still identified the EGFR 19del mutation, and the patient was treated with cisplatin-etoposide for five cycles. Upon further disease progression, lymphadenectomy was conducted, and specimens were collected to establish PDXs (**Figure 1C**). Pathology reconfirmed the diagnosis of transformed SCLC. The patient received four additional cycles of paclitaxel-cisplatin, but the therapy was discontinued due to intolerable adverse effects. Based on the results of the PDX model, anlotinib monotherapy (12 mg q.d., 2 weeks on/1 week off) was initiated, but the disease progressed rapidly, with greatly elevated neuron-specific enolase (NSE) levels (> 370 ng/ml, with a normal range of 0–15 ng/ml). Subsequently, the patient received etoposide (50 mg, q.d., po, 3 weeks on/1 week off) in combination with anlotinib. This treatment led to a substantial decrease in NSE levels to 46.49 ng/ml within one month. However, his overall condition deteriorated with grade 3 hypoproteinemia, limb edema, vomiting, and pain. CT revealed large abdominal and pelvic effusions. The patient passed away in April 2020.

### 2.2 Case 2

**Figure 2:**
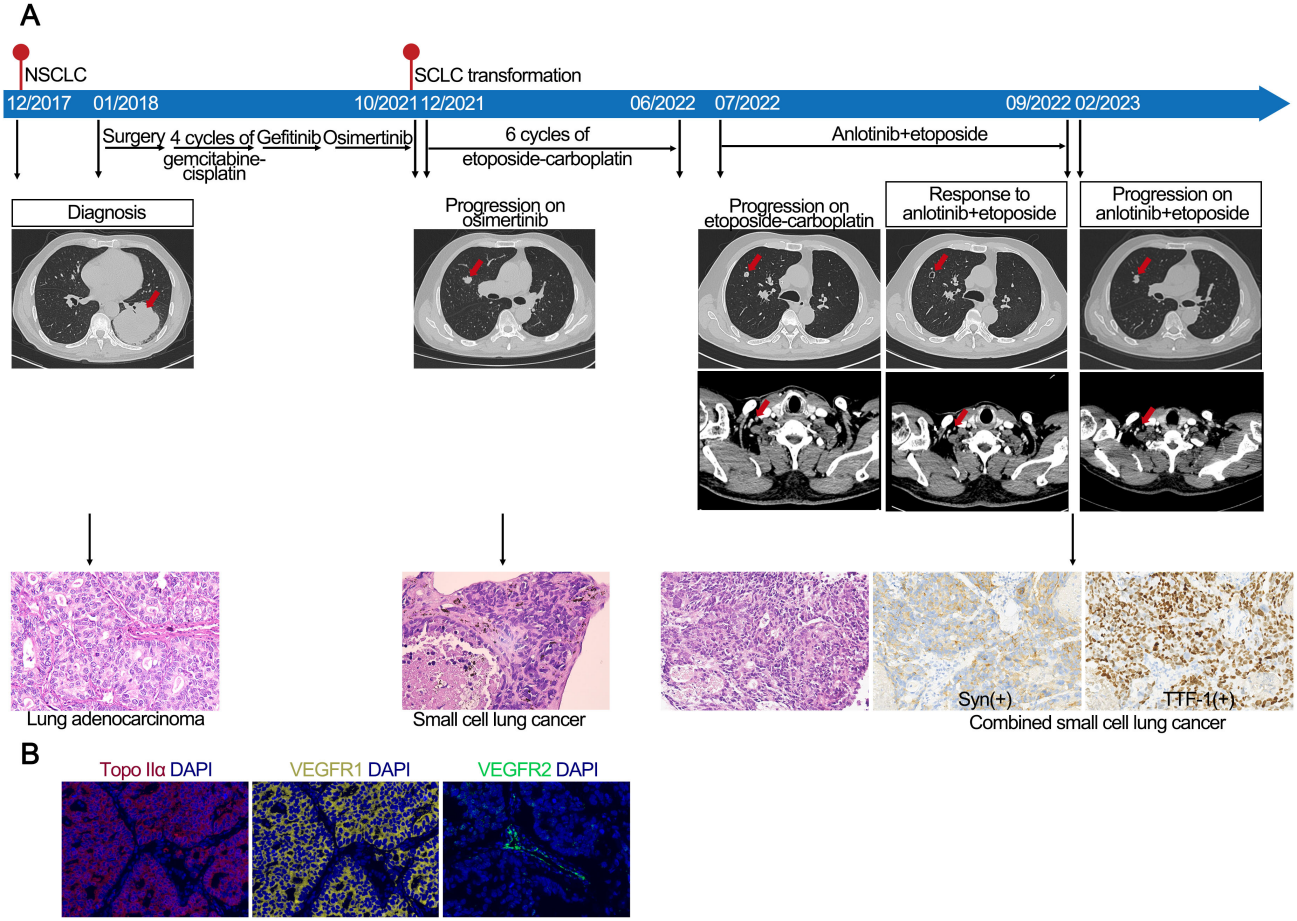
**(A)** Case 2. Case presentation of the transformation of lung adenocarcinoma to small cell lung cancer, including treatment details, available radiographic and pathological findings. **(B)** Immunofluorescence staining of case 2 confirmed the expression of Topo IIα, VEGFR1, and VEGFR2.

Recurrent lung cancer developed in a middle-aged male former smoker with a previous diagnosis of EGFR 19del-mutated LUAD (pT4N1M0, stage IIIA) after curative surgery, followed by four cycles of gemcitabine-cisplatin chemotherapy and 16 months of gefitinib treatment (**Figure 2**). At the time of disease progression, the biopsy confirmed LUAD. NGS identified the EGFR 19del mutation, and the patient began treatment with osimertinib. The osimertinib treatment was continued for 23 months until CT and MRI revealed lung and brain metastases. The biopsy of lung metastasis nodules confirmed the transformation to SCLC. Consequently, the patient received six cycles of etoposide-carboplatin (EC) chemotherapy and cranial irradiation. However, after 7 months of chemotherapy, a CT scan identified metastases in the cervical lymph nodes (LNs) and lung. Then, the patient was treated with etoposide and anlotinib. Two months later, a CT scan showed cavitation in the metastatic lung lesion, and shrinkage of the LN metastases was observed. The patient continued on the etoposide and anlotinib regimen for 7 months until new metastatic lung lesions were discovered. Repeat biopsy showed SCLC combined with LUAD components, which was referred to as combined SCLC (12), with IHC staining positive for synaptophysin and TTF-1.

## 3 Establishment and treatment of the PDX model

Generally, when a patient was diagnosed with SCLC transformation, fresh resection specimens of metastatic LNs were implanted subcutaneously (s.c.) into the right flank of 7- to 8-week-old NOD/SCID mice (P0) purchased from Beijing Vital River Laboratory Animal Technology Co., Ltd. The mice and tumors were monitored for growth for up to 150 days. When the tumor size was >1 cm^3^, PDX tumors were surgically removed and passaged sequentially *in vivo* for tissue expansion. When the tumor size in P2 mice was approximately 130 mm^3^, the mice were randomly divided into four groups: anlotinib alone, etoposide alone, anlotinib plus etoposide, and normal saline (NS) control (n=5). Anlotinib (0.1 mg in 100 μL NS) was administered intragastrically (i.g.) daily for 3 weeks, and 0.2 mg etoposide in 100 μL NS was concurrently intraperitoneally (i.p.) injected on days 1–3 of each week. Mice in the control group received the same volume of NS for i.p. injection and i.g. administration. Tumor volume was measured twice a week and calculated with the formula V = 1/2ab^2^, where a and b represent the length and width of the tumor, respectively. Statistical significance was determined using a *t*-test. A *p*-value <0.05 was considered statistically significant.

## 4 Discussion

Repeated biopsies have recently become more common, leading to an increased identification of SCLC transformation. However, current treatment options remain restricted, and platinum-etoposide is the only feasible and clinically validated effective treatment for transformed SCLC to date (7, 9). In a retrospective study, ten EGFR-TKI-resistant LUAD patients who transformed to SCLC were treated with platinum-etoposide chemotherapy and anlotinib for 4–6 cycles followed by anlotinib maintenance. This regimen achieved a median progression-free survival (mPFS) of 9.0 months and a median overall survival (mOS) of 14.0 months with a favorable safety profile (13). Recently, two cases of SCLC transformation have been reported to be treated effectively with a combination of anlotinib and etoposide. The study provided evidence on the effectiveness of anlotinib combined with oral etoposide in two patients resistant to platinum-etoposide chemotherapy, as well as in the PDX model.

Despite the eventual discontinuation of treatment by the patient in the first case, a significant reduction in NSE levels was observed, and it demonstrated a 7-month PFS in the second case. Therefore, anlotinib combined with oral etoposide served as an effective salvage therapy following the failure of the platinum-etoposide regimen. The immunofluorescence staining of specimens verified the expression of topo IIα and VEGFR, reinforcing the rationale for the adoption of drug regimens. The tumor microenvironment plays essential roles in tumor progression and drug resistance. Indeed, anti-angiogenic drugs have been broadly employed in the clinic (14). Anlotinib is a novel multi-receptor TKI targeting VEGFR, FGFR, PDGFR, and c-kit, effectively inhibiting tumor cell proliferation and tumor angiogenesis (15). It has been reported that anlotinib monotherapy showed promising anti-tumor efficacy in third-line treatment of patients with advanced NSCLC in a randomized phase III trial (ALTER-0303) (10). Additionally, a randomized phase II trial conducted in patients with SCLC highlighted the potential of anlotinib as a third-line or further treatment (11). The complicated microenvironment of SCLC is characterized by poorly differentiated vasculature and immunosuppression (16). The possible mechanism by which the combination therapy of anlotinib and etoposide provided sustained efficacy might be attributed to the capacity of anlotinib to induce a facilitating vessel normalization, improving tumor blood vessel perfusion and alleviating hypoxia, thereby reversing resistance to platinum-based chemotherapy (17, 18). Moreover, in the single-arm phase II study (ACTION-2), the combination of anlotinib with platinum-etoposide chemotherapy achieved an mOS of 19.0 months, supporting the clinical effectiveness of anlotinib plus etoposide in transformed SCLC in our cases (19). Meanwhile, in the randomized phase III trial (ETER701), benmelstobart and anlotinib plus EC achieved an mOS of 19.3 months compared with 13.3 months for anlotinib plus EC, whereas only the benmelstobart-containing regimen provided a significant survival benefit over EC alone (20). Collectively, findings from ACTION-2 and ETER701 suggest that the incorporation of an ICI into the anlotinib plus etoposide regimen might further enhance therapeutic efficacy in transformed SCLC.

Another highlight of the case reports is the application of preclinical models. Leveraging the advantages reported in encapsulating the major complexities of malignancies and studying the heterogeneity of tumors, PDXs can faithfully interpret the concept of personalized treatment. Prior studies have established PDX for transformed SCLC, in which these PDX models are utilized for individually assessing tumor responses to antitumor drugs, exploring potential acquired resistance mechanisms, and investigating subsequent novel effective treatments (21, 22). In this study, we developed a PDX model derived from a patient with transformed SCLC to guide clinical management. This model demonstrated that the efficacy of combined therapy was superior to monotherapy, providing a strong consistency between tumor responses to drug administration in mice and the clinical outcomes in the two patients.

This study has several limitations. First, it reports only two cases, which limits the generalizability of our findings and raises the possibility of patient-specific factors influencing the observed outcomes. Another limitation is the delay between PDX implantation in mice and the treatment schedules for patients. Generally, it takes approximately 2–8 months to establish a PDX model for a preclinical study, which is impractical for patients waiting to commence treatment (23, 24). In case 1, the combination of anlotinib and etoposide reduced the NSE levels and controlled disease progression at the extensive stage. However, the poor overall condition of the patient may have limited the therapeutic benefit of this regimen. Therefore, the use of PDX models in real-time treatment optimization should be carefully considered.

Consequently, the empirical PDX results and clinical findings outlined above provide compelling support for the initiation of further clinical studies on the combined therapy of anlotinib and etoposide.

## 5 Conclusions

In conclusion, this study confirmed the preliminary efficacy and safety of oral etoposide combined with anlotinib in EGFR-TKI-resistant patients with SCLC transformation following the failure of platinum-etoposide chemotherapy. The results of our study provided supportive evidence for the initiation of further clinical studies on the combined therapy. Future research should build on our work to elucidate the therapeutic mechanisms of anlotinib plus oral etoposide in transformed SCLC by using experimental models such as PDXs and to validate its efficacy through prospective clinical trials. Moreover, investigating rational combination strategies, such as incorporating ICIs into the regimen, in future clinical studies may further improve outcomes for patients with SCLC transformation after platinum-etoposide chemotherapy resistance.

## Data Availability

The original contributions presented in the study are included in the article/supplementary material, further inquiries can be directed to the corresponding author/s.

## References

[1] Bray F Laversanne M Sung H Ferlay J Siegel RL Soerjomataram I. Global cancer statistics 2022: GLOBOCAN estimates of incidence and mortality worldwide for 36 cancers in 185 countries. CA Cancer J Clin. (2024) 74:229–63. doi: 10.3322/caac.21834, PMID: 38572751

[2] Travis WD Brambilla E Nicholson AG Yatabe Y Austin JHM Beasley MB. The 2015 world health organization classification of lung tumors: impact of genetic, clinical and radiologic advances since the 2004 classification. J Thorac Oncol. (2015) 10:1243–60. doi: 10.1097/JTO.0000000000000630, PMID: 26291008

[3] Das S Samaddar S. Recent advances in the clinical translation of small-cell lung cancer therapeutics. Cancers (Basel). (2025) 17(2):255. doi: 10.3390/cancers17020255, PMID: 39858036 PMC11764476

[4] Sen T Takahashi N Chakraborty S Takebe N Nassar AH Karim NA. Emerging advances in defining the molecular and therapeutic landscape of small-cell lung cancer. Nat Rev Clin Oncol. (2024) 21:610–27. doi: 10.1038/s41571-024-00914-x, PMID: 38965396 PMC11875021

[5] Giaccone G He Y. Current knowledge of small cell lung cancer transformation from non-small cell lung cancer. Semin Cancer Biol. (2023) 94:1–10. doi: 10.1016/j.semcancer.2023.05.006, PMID: 37244438

[6] Niederst MJ Sequist LV Poirier JT Mermel CH Lockerman EL Garcia AR. RB loss in resistant EGFR mutant lung adenocarcinomas that transform to small-cell lung cancer. Nat Commun. (2015) 6:6377. doi: 10.1038/ncomms7377, PMID: 25758528 PMC4357281

[7] Yin X Li Y Wang H Jia T Wang E Luo Y. Small cell lung cancer transformation: From pathogenesis to treatment. Semin Cancer Biol. (2022) 86:595–606. doi: 10.1016/j.semcancer.2022.03.006, PMID: 35276343

[8] Kim SY Park HS Chiang AC. Small cell lung cancer: A review. JAMA. (2025) 333:1906–17. doi: 10.1001/jama.2025.0560, PMID: 40163214

[9] Marcoux N Gettinger SN O’Kane G Arbour KC Neal JW Husain H. EGFR-mutant adenocarcinomas that transform to small-cell lung cancer and other neuroendocrine carcinomas: clinical outcomes. J Clin Oncol. (2019) 37:278–85. doi: 10.1200/JCO.18.01585, PMID: 30550363 PMC7001776

[10] Han B Li K Wang Q Zhang L Shi J Wang Z. Effect of anlotinib as a third-line or further treatment on overall survival of patients with advanced non-small cell lung cancer: the ALTER 0303 phase 3 randomized clinical trial. JAMA Oncol. (2018) 4:1569–75. doi: 10.1001/jamaoncol.2018.3039, PMID: 30098152 PMC6248083

[11] Cheng Y Wang Q Li K Shi J Liu Y Wu L. Anlotinib vs placebo as third- or further-line treatment for patients with small cell lung cancer: a randomized, double-blind, placebo-controlled Phase 2 study. Br J Cancer. (2021) 125:366–71. doi: 10.1038/s41416-021-01356-3, PMID: 34006926 PMC8329046

[12] Woo JH Kim MY Lee KS Jeong DY Chung MJ Han J. Resected pure small cell lung carcinomas and combined small cell lung carcinomas: histopathology features, imaging features, and prognoses. AJR Am J Roentgenol. (2019) 212:773–81. doi: 10.2214/AJR.18.20519, PMID: 30673331

[13] Ding J Leng Z Gu H Jing X Song Y. Etoposide/platinum plus anlotinib for patients with transformed small-cell lung cancer from EGFR-mutant lung adenocarcinoma after EGFR-TKI resistance: a retrospective and observational study. Front Oncol. (2023) 13:1153131. doi: 10.3389/fonc.2023.1153131, PMID: 37361601 PMC10288518

[14] Altorki NK Markowitz GJ Gao D Port JL Saxena A Stiles B. The lung microenvironment: an important regulator of tumor growth and metastasis. Nat Rev Cancer. (2019) 19:9–31. doi: 10.1038/s41568-018-0081-9, PMID: 30532012 PMC6749995

[15] Shen G Zheng F Ren D Du F Dong Q Wang Z. Anlotinib: a novel multi-targeting tyrosine kinase inhibitor in clinical development. J Hematol Oncol. (2018) 11:120. doi: 10.1186/s13045-018-0664-7, PMID: 30231931 PMC6146601

[16] Li T Qiao T. Unraveling tumor microenvironment of small-cell lung cancer: Implications for immunotherapy. Semin Cancer Biol. (2022) 86:117–25. doi: 10.1016/j.semcancer.2022.09.005, PMID: 36183998

[17] Su Y Luo B Lu Y Wang D Yan J Zheng J. Anlotinib induces a T cell-inflamed tumor microenvironment by facilitating vessel normalization and enhances the efficacy of PD-1 checkpoint blockade in neuroblastoma. Clin Cancer Res. (2022) 28:793–809. doi: 10.1158/1078-0432.CCR-21-2241, PMID: 34844980 PMC9377760

[18] Fan P Qiang H Liu Z Zhao Q Wang Y Liu T. Effective low-dose Anlotinib induces long-term tumor vascular normalization and improves anti-PD-1 therapy. Front Immunol. (2022) 13:937924. doi: 10.3389/fimmu.2022.937924, PMID: 35990640 PMC9382125

[19] Zhang W Deng P Kong T Zhang B Qian F Dong Y. Safety and efficacy of anlotinib in combination with standard chemotherapy as first-line treatment for extensive-stage small cell lung cancer: A multi-center, prospective study (ACTION-2). Lung Cancer. (2022) 173:43–8. doi: 10.1016/j.lungcan.2022.09.003, PMID: 36116169

[20] Cheng Y Chen J Zhang W Xie C Hu Q Zhou N. Benmelstobart, anlotinib and chemotherapy in extensive-stage small-cell lung cancer: a randomized phase 3 trial. Nat Med. (2024) 30:2967–76. doi: 10.1038/s41591-024-03132-1, PMID: 38992123 PMC11485241

[21] Quintanal-Villalonga A Durani V Sabet A Redin E Kawasaki K Shafer M. Exportin 1 inhibition prevents neuroendocrine transformation through SOX2 down-regulation in lung and prostate cancers. Sci Transl Med. (2023) 15:eadf7006. doi: 10.1126/scitranslmed.adf7006, PMID: 37531417 PMC10777207

[22] Chakraborty S Coleman C Manoj P Demircioglu D Shah N de StanChina E. *De novo* and histologically transformed small-cell lung cancer is sensitive to lurbinectedin treatment through the modulation of EMT and NOTCH signaling pathways. Clin Cancer Res. (2023) 29:3526–40. doi: 10.1158/1078-0432.CCR-23-0471, PMID: 37382635 PMC10901109

[23] Yoshida GJ. Applications of patient-derived tumor xenograft models and tumor organoids. J Hematol Oncol. (2020) 13:4. doi: 10.1186/s13045-019-0829-z, PMID: 31910904 PMC6947974

[24] Qu J Kalyani FS Liu L Cheng T Chen L. Tumor organoids: synergistic applications, current challenges, and future prospects in cancer therapy. Cancer Commun (Lond). (2021) 41:1331–53. doi: 10.1002/cac2.12224, PMID: 34713636 PMC8696219

